# Analysis of the Reliability of Feather Sections for Corticosterone Measurement in Pekin Ducks

**DOI:** 10.3390/ani15020138

**Published:** 2025-01-08

**Authors:** Se-Jin Lim, Chan Ho Kim, Ka Young Yang, Woo Do Lee, Su Mi Kim, Yang-Ho Choi, Jung Hwan Jeon

**Affiliations:** 1 Animal Welfare Research Team, National Institute of Animal Science, Rural Development Administration, Wanju 55365, Republic of Korea; limsj818@korea.kr (S.-J.L.); kch8059@korea.kr (C.H.K.); y2k1983@korea.kr (K.Y.Y.); woodo92@korea.kr (W.D.L.); looko@naver.com (S.M.K.); 2 Department of Animal Science, Gyeongsang National University, Jinju 52828, Republic of Korea; yhchoi@gnu.ac.kr; 3 Institute of Agriculture and Life Sciences, Gyeongsang National University, Jinju 52828, Republic of Korea

**Keywords:** feather analysis, corticosterone, ICC, methodology, animal welfare, Pekin ducks, reliability, comparative study

## Abstract

The aim of this study was to identify the feather section, among the whole feather, rachis, and vane, with the highest reliability for corticosterone measurement in 10 Pekin ducks aged 42 days. In total, 60 samples (i.e., 20 per section) were collected and each section was analyzed in duplicate. Corticosterone levels were measured using ELISA and statistical analyses were performed using ANOVA in SAS 9.2, and the intra-class correlation coefficient (ICC) was tested using IBM SPSS. The level in the whole feather (12.55 ± 4.41 pg/mg) was significantly lower (*p* < 0.001) than that in the rachis (18.12 ± 5.70 pg/mg). No significant differences were observed between the rachis and the vane (20.40 ± 3.04 pg/mg). ANOVA results confirmed substantial hormonal variability depending on the feather part analyzed. The ICCs for the whole feather, rachis, and vane were 0.923, 0.876, and 0.004, respectively. The vane section exhibited the highest concentration and lowest variance, whereas the whole feather exhibited the highest ICC. Although the whole feather had the lowest concentration, it exhibited greater consistency. Further research is necessary to improve the feather corticosterone analysis method for more accurate analysis. In conclusion, the whole feather provides the most reliable measure of corticosterone concentration among the three parts.

## Simple Summary

A widely used method for measuring stress in animals is to analyze corticosterone levels in the blood. However, blood sampling can cause pain and stress, which can negatively influence data precision. As an alternative, we measured corticosterone levels in feathers. However, a notable limitation of feather analysis is the variation in hormone levels across different feather sections, which can affect measurement reliability. Therefore, we evaluated the reliability of three feather parts (the whole feather, the rachis, and the vane) to identify the most suitable section for corticosterone assessment. Our results demonstrated that whole feathers provided the most consistent and reliable data despite lower corticosterone concentrations. While the rachis showed higher concentrations, its reliability was lower than that of whole feathers. The vane had the highest concentrations but showed no consistency across the samples. Based on these findings, we recommend using whole feathers to measure stress in individuals. This method offers reliable results with minimal stress to the animals and facilitates the sampling process by allowing the analysis of feathers without separation. These findings could improve the accuracy and efficiency of stress measurements in ducks, especially in field studies, and contribute to enhanced animal welfare.

## 1. Introduction

Stress significantly impacts animal health and productivity, making the development of reliable physiological indicators essential in animal welfare research [1,2,3]. Fluctuations in hormones and nervous system activity are recognized as key tools for assessing stress, offering valuable insights into animal welfare [4,5,6].

Traditionally, blood corticosterone levels have been widely used as an indicator of animal stress. However, this method is invasive and has a notable limitation in being highly sensitive to handling time [7,8,9]. Harvey [10] and Moe [11] reported that the blood sampling process itself could be a major source of stress in animals.

The measurement of corticosterone levels in feathers has been proposed as an alternative [12,13]. The authors suggested that corticosterone present in the bloodstream during feather growth is incorporated into feathers, thereby allowing the assessment of avian stress through feather analysis. Additionally, the stress experienced during feather collection does not affect corticosterone levels in the feathers, making this method superior to blood collection in terms of both animal welfare and experimental reliability.

However, one major limitation of feather corticosterone analysis is the ‘mass-dilution effect’ [14,15]. Corticosterone accumulation may vary depending on the location of the feathers. Bortolotti [12,13] reported that heavier parts of feathers had lower corticosterone concentrations per unit mass than lighter parts. This leads to differences in measurement values across feather sections [14,15].

As mentioned earlier, measuring feather corticosterone is a promising, non-invasive, and long-term stress indicator. However, it is important to address this issue to ensure reliable measurements. Therefore, the aim of this study was to identify the feather section with the highest reliability for stress measurement by determining which part, i.e., the whole feather, rachis, or vane, showed the least inter- and intra-variability.

## 2. Materials and Methods

### 2.1. Animals and Management

From the 2000 ducks reared in the facility, 10 randomly selected 43-day-old Pekin ducks (*Anas platyrhynchos domesticus*) were chosen for sampling. Ducks were housed in open-sided houses under natural ventilation. The animals were reared between September and October 2023. The air temperature ranged from 21 to 29 °C and the relative humidity was 40–61%, confirming that the environmental conditions were suitable and did not induce thermal stress. Ducks were exposed to a natural light/dark cycle throughout the experimental period. Food and water were provided ad libitum (Table 1). The floor was covered with a 25 cm layer of sawdust to ensure appropriate bedding conditions. The ducks were maintained at a stocking density of 3.6 birds/m^2^. All ducks were visually inspected for signs of disease or injury, and no abnormalities were observed throughout the experimental period.

### 2.2. Measurements and Analysis

#### 2.2.1. Feather Collection

Twenty feathers were collected from the scapular region by cutting them close to the skin [16,17,18]. We used clean feathers, excluding biological fouling, such as feces or blood.

Each section was analyzed in duplicate, using 6 feather samples per individual, totaling 60 samples (i.e., 20 samples per whole feather, rachis, and vane). The amount of corticosterone accumulated in feathers varies according to their growth rate, which differs depending on the type of feather (e.g., body feathers vs. flight feathers). Therefore, morphologically similar features were considered [13].

**Figure 1:**
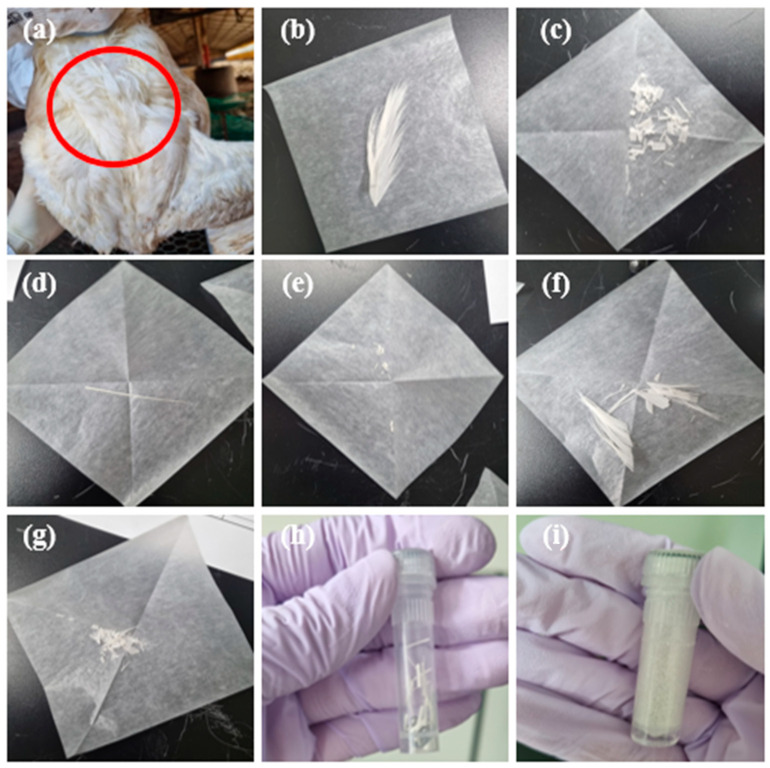
Photograph of feather samples showing the processing steps. (**a**) Feather sampling site (scapular region), (**b**) before mincing the whole feather, (**c**) after mincing the whole feather, (**d**) before mincing the rachis section, (**e**) after mincing the rachis section, (**f**) before mincing the vane section, (**g**) after mincing the vane section, (**h**) before pulverization, and (**i**) after pulverization.

All samples had the same morphology and a similar total weight (15 ± 8 mg) (Figure 1). Feather samples were collected on the same day, wrapped in foil to eliminate light effects, and stored in plastic bags at room temperature (23–26 °C).

#### 2.2.2. Feather Sample Preparation

Twenty feathers were prepared and assayed following the method in [16,17], with some modifications. The effect of methanol volume or additional extractions on corticosterone recovery was not tested in this study. Twenty feathers were placed in a 50 mL conical tube, washed once with distilled water, washed with 70% ethanol, and then washed again with distilled water. The washed samples were then dried in an airflow hood for 12 h. Six feathers per individual were selected based on morphology and a weight of ≥0.1 g (Table 2).

#### 2.2.3. Assay Validation

Testing the precision of the assay used to measure biological variables is essential to performing an accurate analysis [19]. The assay was validated based on recommendations from previous studies [18,20,21]. The precision of the ELISA was proven by the intra- and inter-assay coefficients of variation and the linearity of dilution. The coefficient of variation (CV) was run twice per assay, whereas the actual samples underwent a single run per assay.

##### Coefficient of Variation

Intra- and inter-assay CVs were measured to test the precision of the ELISA. Six different samples at three concentration levels (low, medium, and high) were used, with two replicates for each concentration to measure the CV [22,23]. The assays were performed in duplicate to measure the inter-assay CV. The intra- and inter-assay CVs were evaluated using 12 and 24 samples, respectively. The data were valid with intra- and inter-assay CVs of 8.9% and 19.8%, respectively [24].

##### Dilution Linearity

The dilution linearity was examined to test the specificity of the ELISA. In this process, a 200,000 pg/mL sample was diluted 1:2 and analyzed using five concentrations (12,500, 6250, 3125, 1562, 781, and 390 pg/mL) within the standard range of the ELISA kit (*R*^2^ value above 0.99). These results revealed that ELISA was feasible for the analysis of feather corticosterone.

### 2.3. Feather Corticosterone

Corticosterone levels were expressed as pg/mg [25,26], except for in dilution linearity. Corticosterone concentration in the feather samples was measured using an enzyme-linked immunosorbent assay (ELISA) kit (cat no. ADI-900-097; Enzo Life Sciences, Farmingdale, NY, USA), and the absorbance was measured at 405 nm using an ELISA reader (Thermo Fisher Scientific, Waltham, MA, USA).

### 2.4. Statistics

All data followed a normal distribution (*W* = 0.99, *p* = 0.98), as verified using the Shapiro–Wilk test in SAS 9.4. We calculated the intra-class correlation coefficient (ICC) based on a two-way random effects model using IBM SPSS and Bland–Altman plots to assess reproducibility. ANOVA was used to determine significant differences in corticosterone levels among feather sections, and Tukey’s test was used for post hoc comparisons (α = 0.05).

Whole feather samples were cut into small pieces no longer than 3 mm using surgical scissors. The rachis and vane samples were separated and cut into small pieces. The finely chopped feather samples were transferred into tubes containing beads (TacoPrep Preloaded Steel Bead Tubes, GeneReach, Taichung City, Taiwan) and ground into powder using a beadbeater (Taco Prep Bead Beater, GeneReach) for 12 min.

After grinding, the feather samples were adjusted to the nearest 10 mg (10 ± 0.01 mg). Then, 1.5 mL of 99.5% methanol was added to each sample and the mixture was vortexed at room temperature for 30 min. The samples were then transferred to a shaking incubator and incubated at 37 °C for 17 h at 200 rpm.

The samples were centrifuged at 3500× *g* for 15 min. From the resulting supernatant, 1 mL was transferred to a 1.5 mL tube and dried in an oven at 37 °C until all the liquid evaporated. Subsequently, 0.25 mL of assay buffer provided in the ELISA kit was added to the residue, and the mixture was vortexed for 1 min. The samples were then stored at −20 °C until ELISA analysis.

## 3. Results and Discussion

### 3.1. Whole Feather

We conducted a Bland–Altman (BA) plot and ICC analysis to evaluate the within-individual variation in whole feathers. For assessing within-individual variation, two measurements were taken for each individual, from the whole feather and the rachis and vane sections. The BA plot was used to assess the agreement between the two measurements and visualize the data distribution. This plot can also be used to assess the repeatability, reproducibility, and acceptability of a new method compared to a standard reference [16,27,28,29,30,31,32,33]. Following the method in [30,34], we plotted the average of the two measurements on the *x*-axis and the difference between them on the *y*-axis, with the mean difference as the middle horizontal line. The upper and lower limits of agreement (LOAs) were calculated as the mean difference ± 1.96 times the standard deviation.

**Figure 2:**
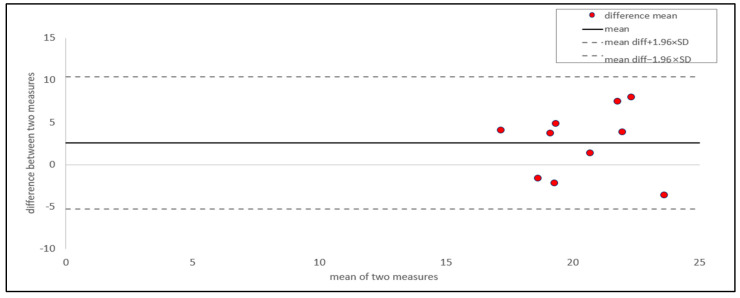
Bland–altman plot of corticosterone in the whole feather part. Red points represent the differences between the two measurements taken from two feathers per individual, plotted against their mean (*n* = 10). The solid horizontal line indicates the mean difference between the two measurements. The dashed horizontal lines represent the 95% limits of agreement, calculated as the mean difference ± 1.96 times the standard deviation of the differences.

The BA plot (Figure 2) showed that the average difference between the two measurements of the whole feather was 2.49, with a 95% confidence interval (CI) ranging from −2.18 to 7.16. Most values were concentrated around the mean line, and all data points fell within the 95% CI. This indicated a high degree of agreement between the two measurements. Dogan [30] and Giavarina [34] reported that an ideal model would show that measurements obtained using different methods produce identical results, with all differences equal to zero. If the mean difference is a positive or negative value, it indicates the extent of the discrepancy between the two measurements by that amount [29,30,35]. However, as stated by Park [36] and Muller [37], it is unreasonable to expect identical results when measuring biological phenomena. Thus, it is more practical to focus on the extent of differences rather than expecting whole consistency. As the 95% CI of the BA plot included zero and independent t-tests and showed no significant differences among the three parts, the slightly higher average values were marginal and negligible [35,38,39]. The intra-class correlation coefficient (ICC), also known as the reliability coefficient, is a commonly used metric for assessing repeatability and reproducibility [40]. The ICC, which reflects the proportion of total variance that is not due to measurement error, is widely used to assess data reliability [33,37,41,42,43,44,45]. Based on the approaches of Kong [29] and Han [45], we performed an ICC analysis (two-way random effects model, absolute agreement, and mean rating) to assess the reliability between the two measurements (Table 3). The whole feather showed a high ICC (0.92; 95% CI: 0.69–0.98), indicating a high level of consistency [46,47,48,49].

This suggested that the whole feather was more consistent across samples than the other parts (rachis and vane), with less between-individual variability. As shown in Table 3, the whole feather showed the highest ICC (0.92; 95% CI: 0.69–0.98) among the three parts, reflecting a very high value. This indicated excellent reliability [46,47,48,49]. This result suggested that the whole feather provides more consistent measurements across samples than the other parts (rachis and vane), with less between-individual variability. Although the rachis also exhibited a high ICC (0.88), the 95% CI for the whole feather was narrower than that of the rachis around the ICC value. This confirmed that the whole feather provides the most reliable measurement of the three parts [38,45].

**Figure 3:**
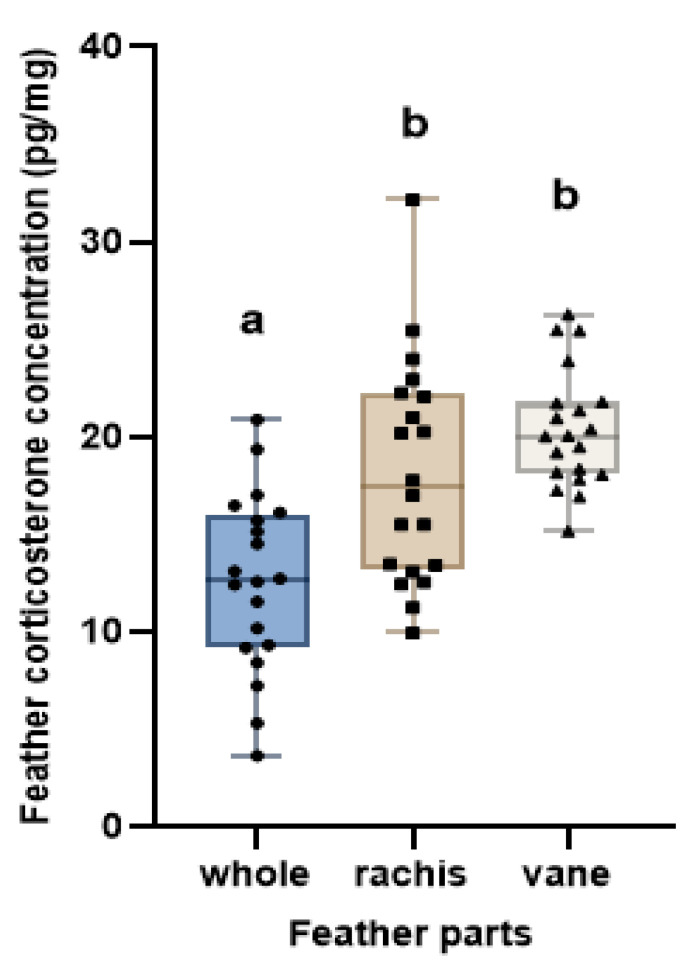
Corticosterone concentration (pg/mg) of the whole feather, rachis, and vane. Each point (circle, square, or triangle) represents an individual measurement of the respective feather parts. The horizontal line within each box indicates the median concentration. Boxes represent the interquartile range (IQR), and whiskers denote the data range, excluding outliers. a,b values above the box plot indicate a statistically significant difference (Tukey’s HSD test, p < 0.05).

Corticosterone concentrations differed significantly across feather sections (Figure 3), which is consistent with findings from a previous study [15]. The whole feather group exhibited significantly lower concentrations than the other two groups (Figure 3; *F* = 15.69, *p* < 0.0001). Given that there were no differences in experimental conditions, we concluded that these variations were due to physical differences in the feather samples. This is likely due to the heterogeneous composition of the whole feather, which includes different physical structures such as the rachis and vane [14,50]. This makes it difficult to achieve sufficient pulverization, leading to a less uniform reduction in particle size. As a result, the reduced surface area induces a low extraction efficiency. Particle size reduction enhances the surface area accessible for mass transfer, subsequently increasing the extraction yield [15,51,52,53].

According to Mohamed [53], a more homogeneous and finely ground feather matrix improves extraction yield and reliability of the assessment. Additionally, homogenization of ground feather has been shown to improve the reliability of cortisol measurements by increasing the proportion of corticosterone extracted.

Although the corticosterone concentration in the whole feather (12.55 ± 4.53 pg/mg) was significantly lower than that in other sections, it showed the most symmetrical distribution around the mean (Figure 3). This indicated that the whole feather had the lowest variability among the three sections, consistent with the BA plot and ICC analysis results. Our study prioritized consistency and reliability at high concentrations. Therefore, despite its lower concentration, the whole feather provided the most consistent data with the highest within-individual reliability, suggesting that it is the most appropriate indicator for stress measurement.

### 3.2. Rachis

**Figure 4:**
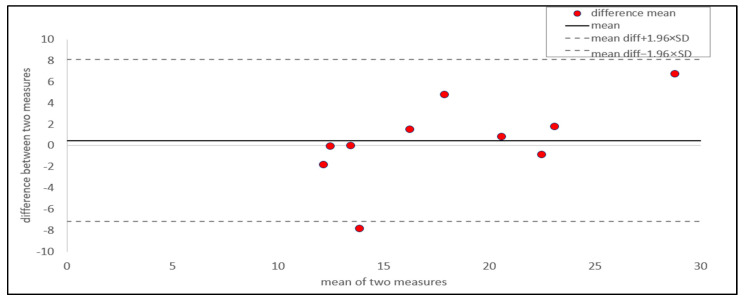
Bland–altman plot of corticosterone in rachis part. Red points represent the differences between the two measurements taken from two feathers per individual, plotted against their mean (*n* = 10). The solid horizontal line indicates the mean difference between two measurements. The dashed horizontal lines represent the 95% limits of agreement, calculated as the mean difference ± 1.96 times the standard deviation of the differences.

The BA plot (Figure 4) shows that the mean difference for the rachis was 0.48, with a 95% CI ranging from −7.16 to 8.12. Except for one outlier, all values fell within the 95% CI. The data showed a uniform distribution of measurements within the CI, regardless of the magnitude or direction (positive or negative) of the values. This indicated that the disagreement between the two measurements was unrelated to the size of the values, confirming that the analysis was conducted properly without errors [29,30,34,35,36,54].

The ICC for the rachis (0.88) was lower than that for the whole feather (0.92) (Table 3). However, the mean difference for the rachis (0.48) was less than that for the whole feather (2.49). The closer the mean difference is to zero, the higher the agreement between measurements [35]. The results of this study showed that although the difference between the two measurements for the rachis was small, the overall data for the rachis exhibited greater variability than that for the whole feather. The BA plot also showed that the data for the rachis were more widely distributed than those for the whole feather, with a broader CI. Therefore, we found that the rachis had a lower measurement reliability than the whole feather.

Corticosterone concentrations differed significantly between feather sections, although no significant differences were found between those in the rachis and the vane. The rachis (18.12 ± 5.70 pg/mg) exhibited the greatest variability among the three parts. This variability is likely related to the methods used to process the rachis.

Previous studies have reported that the vane contains significantly higher corticosterone per unit mass than the rachis, owing to the mass-dilution effect [12,13,15,21]. However, we did not detect a significant difference in corticosterone concentrations between the rachis and vane (Figure 3). This difference was considered to be due to variations in sample preparation methods. In our study, feathers were ground for 12 min, whereas other studies followed different protocols. Nikole [15] ground the samples into a homogenous powder and Haffelin [21] minced them into pieces smaller than 5 mm. These methodological differences in feather processing may have induced variations in the results between studies. There were no significant differences between the rachis and the vane in this study (Figure 3). This is consistent with previous research [14,15] indicating that grinding feathers into a fine, homogenous powder reduces structural variation and largely eliminates the mass-dilution effect.

Ntalikwa [55] reported that particle size affects the extraction efficiency and that the optimal particle size depends on the solvent and solute properties. He stated that the particle size was too small and that agglomeration among the particles could reduce the surface area available for solvent contact, thereby decreasing the extraction efficiency. Previous studies have shown that the grinding process affects the rachis more than the vane owing to its dense and rigid structure. Thus, the degree to which the rachis was evenly chopped before grinding and the uniformity of the powder across the samples likely contributed to the observed variability.

However, the high ICC and concentration observed in the rachis suggested that it could serve as a valuable indicator for analysis. If the methodology is further optimized, the rachis could become a suitable part of feather analysis.

### 3.3. Vane

The vane part (20.40 ± 3.04 pg/mg) showed higher corticosterone concentration than the whole feather part and the narrowest data distribution (Figure 3), suggesting that the vane exhibited higher consistency than the other sections. Initially, we hypothesized that the vane would have the highest reliability because of its low variability within the higher concentration range, but its reliability was very low.

**Figure 5:**
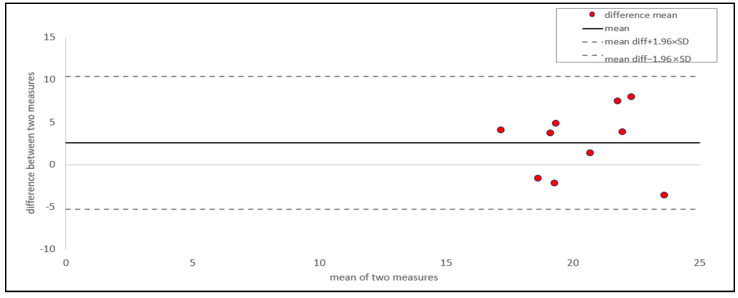
Bland–altman plot of corticosterone in vane part. Red points represent the differences between the two measurements taken from two feathers per individual, plotted against their mean (*n* = 10). The solid horizontal line indicates the mean difference between two measurements. The dashed horizontal lines represent the 95% limits of agreement, calculated as the mean difference ± 1.96 times the standard deviation of the differences.

According to the BA plot (Figure 5), the mean difference for the vane was 2.55, with a 95% CI ranging from −5.26 to 10.36. Although all the data points fell within the 95% CI, the vane displayed the widest confidence interval and the most dispersed data among the three sections. Because the BA plot showed differences between the two measurements, this pattern indicated that the vane had high within-individual variability [25,35,38].

The reliability analysis further substantiated these results. The ICC analysis showed that the vane had a very low reliability (ICC = 0.00; 95% CI: −3.01–0.75, *p* = 0.498), indicating poor reproducibility and low within-individual consistency [46,47,48,49]. This result confirmed that the vane section exhibited high within-individual variability.

In contrast to the vane (ICC = 0.00), the whole feather (ICC = 0.92) and rachis (ICC = 0.88) exhibited high reliability (Table 3). These results were presumably due to the unique properties of the vane, such as uneven hormone accumulation and inconsistent corticosterone extraction. If corticosterone accumulation from the rachis to the vane differed across feathers, it could have contributed to increased within-individual variability. The distribution of melanin within vanes has also been studied [56,57]. However, the pattern of corticosterone accumulation within the vanes has not yet been explored.

Because feathers with similar morphology and size collected from the same individual reflect the same timeline, circulating corticosterone levels in the bloodstream during feather growth are also identical [13,14]. However, high variability within individuals suggests that the degree of accumulation from the rachis to the vane may differ. If hormones are not uniformly deposited within the vane, variability increases, resulting in lower reliability.

Because the pattern of corticosterone deposition within feathers has not yet been clearly identified, various approaches to analyzing feather corticosterone concentrations have been proposed, including mass-dependent [21,58,59], time-dependent [12,13,60,61], and chemical-composition-based methods [62,63]. Further studies are needed to determine the pattern of corticosterone deposition in feathers and to determine corticosterone concentrations more accurately.

The vane (20.40 ± 3.04 pg/mg) exhibited higher corticosterone concentration than whole feather part, though no significant difference was found between the vane and rachis. The vane showed the lowest within-individual reliability (ICC = 0.00), and its consistency across samples was poor. As the objective of this study was to identify the section with the highest analytical reliability, we concluded that the vane was not appropriate for feather analysis despite its high concentration.

## 4. Conclusions

We conducted a BA plot and ICC analysis to evaluate the reliability of the corticosterone analysis using feather sections. The whole feather (ICC = 0.923; 95% CI: 0.691–0.981) and the rachis (ICC = 0.875; 95% CI: 0.499–0.969) demonstrated high reliability, whereas the vane (ICC = 0.004; 95% CI: −3.011–0.753) exhibited very low reliability. The ANOVA results revealed no significant differences in corticosterone concentrations between the rachis and vane. However, the whole feather (12.55 ± 4.53 pg/mg) showed significantly lower concentrations compared with both the rachis (18.12 ± 5.70 pg/mg) and the vane (20.40 ± 3.04 pg/mg).

Although the rachis had a slightly lower reliability than the whole feather, its higher corticosterone concentration suggests that this allowed for more effective extraction and detection. Thus, it could serve as a reliable indicator for feather analysis if methods to reduce the variation in the rachis can be developed. However, despite having the highest concentration, the vane exhibited extremely low reliability. This renders it unsuitable for analysis. Although the whole feathers exhibited the lowest corticosterone concentrations, they showed the highest reliability. Additionally, they offer practical advantages during analysis because they does not require additional steps to separate the other parts. Nevertheless, due to the low corticosterone concentration in the whole feather, future studies should focus on ways to increase the sensitivity of the method without sacrificing reliability. Considering these results, whole feathers appear to be the most appropriate method for evaluating individual stress levels through feather analysis.

## Figures and Tables

**Table 1:** Composition and nutrient content of diets for ducks.

Ingredient (%)		Starter Period	Finisher Period
		(0–2 Week)	(3–6 Week)
Corn		54.55	58.90
Wheat bran		2.50	14.60
Soybean meal		37.70	15.35
Corn gluten meal		1.50	7.00
Soybean oil		0.50	1.00
Limestone		0.45	0.70
Dicalcium phosphate		1.40	1.00
DL-Methionine		0.10	0.05
L-Lysine		0.05	0.05
Mineral mixture		1.00	1.00
Salt		0.25	0.25
Total		100.00	100.00
Chemical composition	ME (kcal/kg)	2945	3016
	CP (%)	22.40	18.40
	Methionine (%)	0.44	0.39
	Lysine (%)	1.27	0.86
	Ca (%)	0.76	0.66
	P (%)	0.46	0.35

**Table 2:** Overview of the measured results.

		Weight of Feathers (mg)	Final Feather Weight (mg)	Corticosterone in Feather (in pg/mg)
Whole	Mean	16.28	10	12.55
	Max	27	10	20.88
	Min	8	8	3.64
Rachis	Mean	7.3	10	18.12
	Max	11.8	12	32.16
	Min	3.6	10	9.95
Vane	Mean	21.55	10	20.40
	Max	38.2	10	26.28
	Min	8.2	8	15.17

**Table 3:** Intra-class correlation coefficients for measurement consistency.

Sections	ICC	95% CI		*p*-Value	Model Type
		Lower	Upper		
Whole	0.92	0.69	0.98	<0.001	Two-way random
Rachis	0.88	0.50	0.97	0.002	Two-way random
Vane	0.00	−3.01	0.75	0.498	Two-way random

## Data Availability

The original contributions presented in the study are included in the article.
